# Neurologists-level interpretable CT-based deep neural network for prediction of hemorrhagic transformation after ischemic stroke

**DOI:** 10.3389/fnins.2025.1753071

**Published:** 2026-01-14

**Authors:** Guanyi Zhang, Yanrui Jin, Mengxing Wang, Xu Han, Yihui Tu, Zixiao Li, Xingquan Zhao, Qian Zhang

**Affiliations:** 1 Department of Neurology, Beijing Tiantan Hospital, Capital Medical University, Beijing, China; 2 China National Clinical Research Center for Neurological Diseases, Beijing, China; 3 School of Mechanical Engineering, Shanghai Jiao Tong University, Shanghai, China; 4 Department of Neurology, Guangrao People Hospital, Dongying, Shandong, China; 5 Department of Mathematics, Shanghai University, Shanghai, China; 6 Research Unit of Artificial Intelligence in Cerebrovascular Disease, Chinese Academy of Medical Sciences, Beijing, China

**Keywords:** assisted decision making, computed tomography, deep neural network, hemorrhagic transformation, prediction, stroke

## Abstract

**Background:**

Hemorrhagic transformation (HT) is a severe complication following acute ischemic stroke, associated with neurological deterioration and poor clinical outcomes. Deep learning represents a promising tool for HT prediction.

**Methods:**

We conducted a retrospective analysis of 474 acute ischemic stroke cases (231 HT and 243 non-HT) admitted to Beijing Tiantan Hospital from April 2014 to November 2022. We constructed a dataset from this cohort and randomly partitioned it into training and validation sets. Subsequently, we developed a model utilizing convolutional neural networks (CNNs) and residual networks based on computed tomography (CT) scans to predict HT after ischemic stroke.

**Results:**

The final dataset consisted of 613 CT scans. The model achieved an F1 score of 78.94% (95% CI, 67.7–86.4). The Area Under the Curve (AUC) was 0.842 (95% CI, 75.8–92.1), sensitivity was 71.55% (95% CI, 60.6%−85.0%), and accuracy was 74.52% (95% CI, 63.9%−83.2%).

**Conclusion:**

By combining plain CT scans with deep learning methodologies, we developed a clinically applicable model with demonstrable interpretability. Primarily designed to predict HT after acute ischemic stroke, this model demonstrated significant performance advantages in testing compared to both clinical physicians and similar existing models.

## Introduction

Stroke is a devastating condition associated with high risks of disability, mortality, and recurrence, posing a significant socioeconomic burden (Liu et al., 2020). Over the past several decades, the global burden of stroke has continued to rise, driven by population growth, aging demographics, and the increasing prevalence of risk factors (Katan and Luft, 2018).

Hemorrhagic transformation (HT) after ischemic stroke is defined as the detection of bleeding on a follow-up computed tomography (CT) or magnetic resonance imaging (MRI) scan in patients with no evidence of hemorrhage on the initial post-infarction scan, or hemorrhagic infarction identifable on the initial scan (Chinese Society of Neurology Chinese Stroke Society, 2019). HT is a critical concern in the management of acute ischemic stroke and represents part of the natural history of cerebral infarction (Moulin et al., 1994). The clinical incidence of HT ranges from 2.2% to 44.0%, while the pathological incidence can reach up to 70% (Aviv et al., 2009). Current treatment options for ischemic stroke, including intravenous thrombolysis, endovascular therapy, anticoagulants, and antiplatelets (Mendelson and Prabhakaran, 2021), carry the risk of increasing the frequency and severity of HT (Moulin et al., 1994). Consequently, HT contributes significantly to the underutilization of reperfusion therapies and is associated with poor prognosis (Yaghi et al., 2015). Studies indicate that HT increases the risk of death by 8- to 10-fold (Yen et al., 2016), with evidence suggesting a higher bleeding propensity in Asian populations compared to Western populations (Shen et al., 2007; Ueshima and Matsuo, 2002; Kim et al., 2015). While symptomatic HT has garnered significant attention, the significance of asymptomatic HT remains controversial (Hacke et al., 2004; Berger et al., 2001; Albers et al., 2006; Molina et al., 2002; von Kummer, 2002; Dzialowski et al., 2007), despite its association with unfavorable long-term cognitive and neurological outcomes (Lei et al., 2014; Dzialowski et al., 2007).

Neuroimaging remains the principal method for predicting HT. Numerous studies have attempted to predict HT using non-contrast imaging signs, such as the hyperdense artery sign (Strbian et al., 2012), leukoaraiosis (Whiteley et al., 2012), collateral circulation (Bang et al., 2011), and hyperintense acute injury markers (Kidwell et al., 2008). Traditionally, logistic regression was the standard for analyzing prognostic data (Asadi et al., 2014). However, the emergence of machine learning and deep learning algorithms has demonstrated superior potential in outcome prediction and clinical decision support (Zihni et al., 2020; Khera et al., 2021). For instance, James et al. successfully employed support vector machines (SVM) to predict symptomatic HT following thrombolysis (James et al., 2018).

CT is an efficient, widely available diagnostic tool and serves as the primary imaging modality for acute ischemic stroke (Vilela and Rowley, 2017). In many resource-limited settings, non-contrast CT (NCCT) is often the sole imaging option available for initial evaluation. Given the variability in onset-to-visit times, we conducted a retrospective study using NCCT images obtained at various time points. Our study aims to present a novel deep-learning approach for predicting post-stroke HT using NCCT. This tool is intended to assist emergency neurologists in making informed medication decisions for high-risk patients.

## Methods

**Figure 1:**
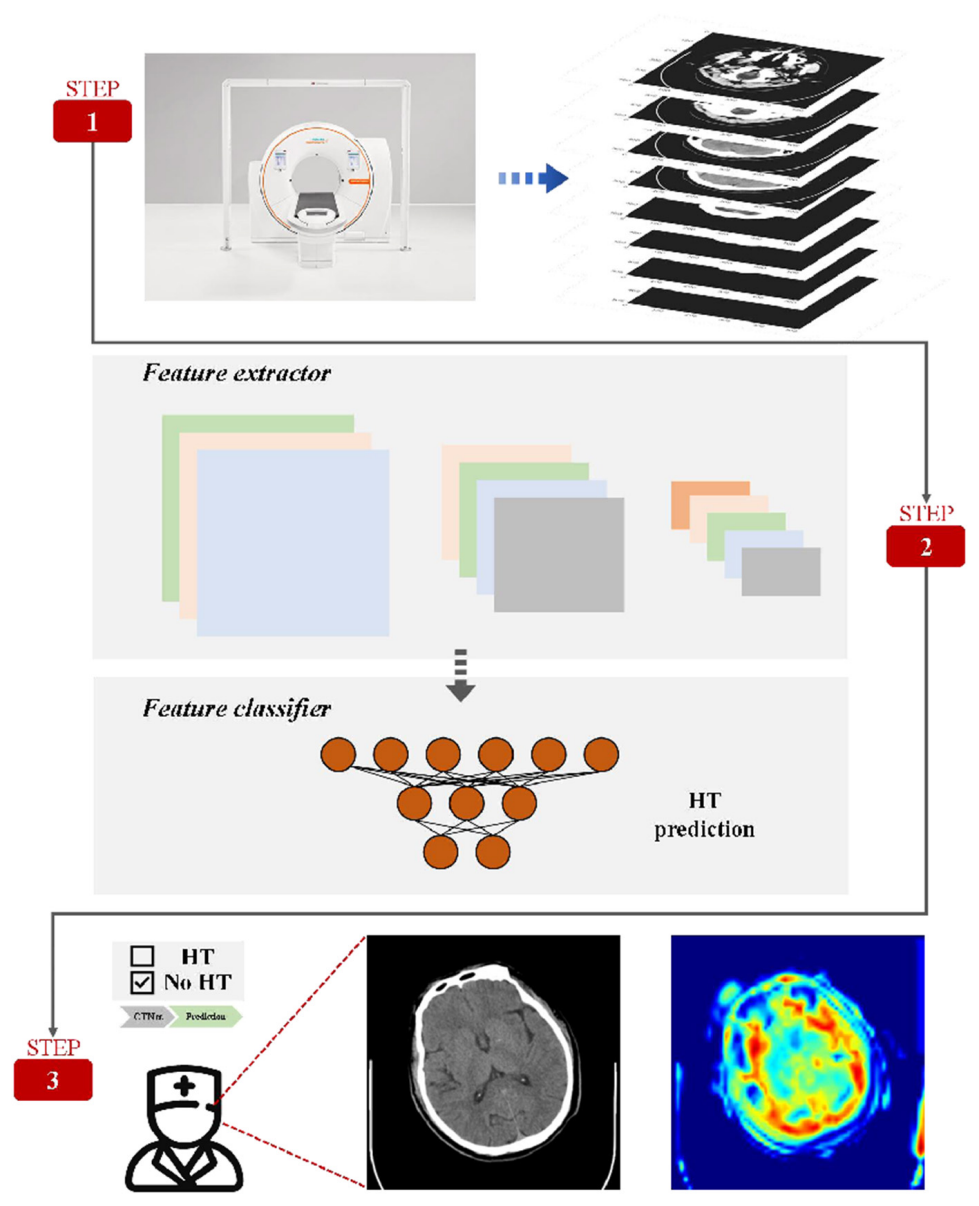
Method framework. A complete set of CT is given by the data preprocessing method. The model enables automatic prediction of HT and automatic selection of areas of interest useful for diagnosis.

Figure 1 describes this prediction system in detail. The prediction system consists convolutional neural network (CNN) and Residual blocks, which are used to describe the deep characteristics of CT.

### Dataset and partition

We retrospectively analyzed 474 patients with ischemic stroke admitted to Beijing Tiantan Hospital between April 2014 and November 2022, collecting both demographic and imaging data. HT was defined as the absence of bleeding on the initial CT/MRI scan, followed by the detection of bleeding on a subsequent scan. Patients were included only if their initial scan showed no evidence of HT and they underwent serial imaging during hospitalization.

Screening was based on clinical and imaging diagnoses reviewed by researchers; discrepancies were adjudicated by a three-expert committee. To reflect clinical reality, multiple images from the same patient (prior to HT onset) were incorporated. We included non-HT patients in a nearly 1:1 ratio, matched by age and gender. The dataset was randomly partitioned into a training set and a test set at a 9:1 ratio. Additionally, eight specialized neurologists were enlisted to evaluate the imaging data in the validation set. Blinded to clinical information and relying solely on NCCT images (conditions identical to the model), the clinicians predicted the probability of HT based on their experience. These predictions were subsequently compared with the model's output.

### Data pre-processing

CT data were collected with a fixed layer thickness of 5 mm. However, due to individual and machine variations, the number of layers differed. We cropped the CT images to maintain a standardized frame count dimension of 28 for each input (Supplementary figure1).

### CNN and residual block

CNN is presented and further explored by Fukushima (1980) and Lecun et al. (1998). Due to the strong representative capability, CNN has been applied in different fields, such as mechanical metamaterial design (Bonfanti et al., 2020), biomedical application (Kusumoto et al., 2021; Green et al., 2022), and all-cause mortality prediction (Ulloa Cerna et al., 2021). However, some researchers (He et al., 2016) found an idea that the increasing of CNN layers cannot continuously improve the model performance but decrease the model performance. Thus, inspired by the insight of the article (He et al., 2016), we design different residual blocks for extracting deep features. The details of residual learning process are listed in the Equations 1, 2.


$$\begin{array}{l} Y_{L,1}=F\left(X_{L},W_{L},b_{L}\right)+X_{L} \end{array}$$ (1)

$$\begin{array}{l} Y_{L,2}=F\left(X_{L},W_{L},b_{L}\right)+{H(X}_{L}) \end{array}$$ (2)

Where X represents input vectors, F(.) means the nonlinear mapping function and H(.) represents the linear shortcut function.

Supplementary Figure 2 shows the designed different residual blocks. Residual block1 is used to process the input whose dimension is consistent with the output dimension. Additionally, Residual block2 is used to process the input whose dimension is inconsistent with the output dimension. To sum up, we apply CNNs and residual blocks for extracting deep characteristic of CT information for modeling HT prediction system.

### The architecture

Supplementary Figure 3 lists the architecture of the proposed CTNet, which includes CNN layer, residual blocks and HT predictor.

In CT data, the forms and positions of lesions in each layer of images are different, and subtle changes will affect the final prediction results. In this paper, CNN and residual blocks are used to automatically extract complex deep features and realize end-to-end recognition of CT image to HT prediction results. The pre-processed CT data is directly input into the CNN layer to extract the shallow layer information and increase the dimension of features. Then, two kinds of residual blocks designed in this paper are stacked in sequence to continuously extract deep features and ensure that model performance does not degrade. Finally, HT predictor is used to further process the deep features and get the prediction results.

### Statistical approach

All statistical analyses were performed using Python (V3.8). We compared accuracy, sensitivity, positive predictive value (PPV), specificity, F1 score and Area Under the Curve (AUC) between the model and the eight neurology specialists. The calculation methods for the model evaluation metrics are as follows:


$$\begin{array}{l} Accuracy=\frac{TP+TN}{TP+TN+FP+FN} \end{array}$$ (3)

$$\begin{array}{l} Sensitivity=\frac{TP}{TP+FN} \end{array}$$ (4)

$$\begin{array}{l} PPV=\frac{TP}{TP+FP} \end{array}$$ (5)

$$\begin{array}{l} Specificity=\frac{TN}{TN+FP} \end{array}$$ (6)

$$\begin{array}{l} F1=\frac{2\times Sen\times PPV}{Sen+PPV} \end{array}$$ (7)

## Results

Table 1 lists the details of the dataset. The cohort included 474 patients [mean age 62.45 ± 11.85 years; 123 (25.95%) females]. Treatment modalities included thrombolysis (101, 21.31%), antiplatelet therapy (314, 66.24%), and anticoagulation (12, 2.53%). The dataset contained 611 CT images acquired at various time points, comprising 243 patients in the HT group and 231 in the non-HT group. The training set included 437 patients (548 images), while the test set included 61 patients (61 images).

**Table 1:** Baseline of dataset.

**Characteristic**	**Total set (*n =* 474)**	**Training set (*n =* 437)**	**Test set (*n =* 61)**
Age, years	62.45 ± 11.85	62.47 ± 11.70	63.33 ± 13.13
**Sex (** ***N*** **, %)**			
Male	351 (74.05)	323 (73.91)	47 (77.05)
Female	123 (25.95)	114 (26.09)	14 (22.95)
**Treatment (** ***N*** **, %)**			
Antiplatelet agent	314 (66.24)	288 (65.9)	44 (72.14)
Anticoagulant agent	12 (2.53)	12 (2.75)	0 (0.00)
Thrombolytic agent	101 (21.31)	94 (21.51)	9 (14.75)
Not using the above drugs	41 (8.65)	37 (8.47)	8 (13.11)
Treatment not available	6 (1.27)	6 (1.37)	0 (0.00)

*N* is the value after removing duplicates. Train set (*n* = 437) + Test set (*n* = 61) ≠ Total set (*n* = 474).

### Testing and performance evaluation

**Figure 2:**
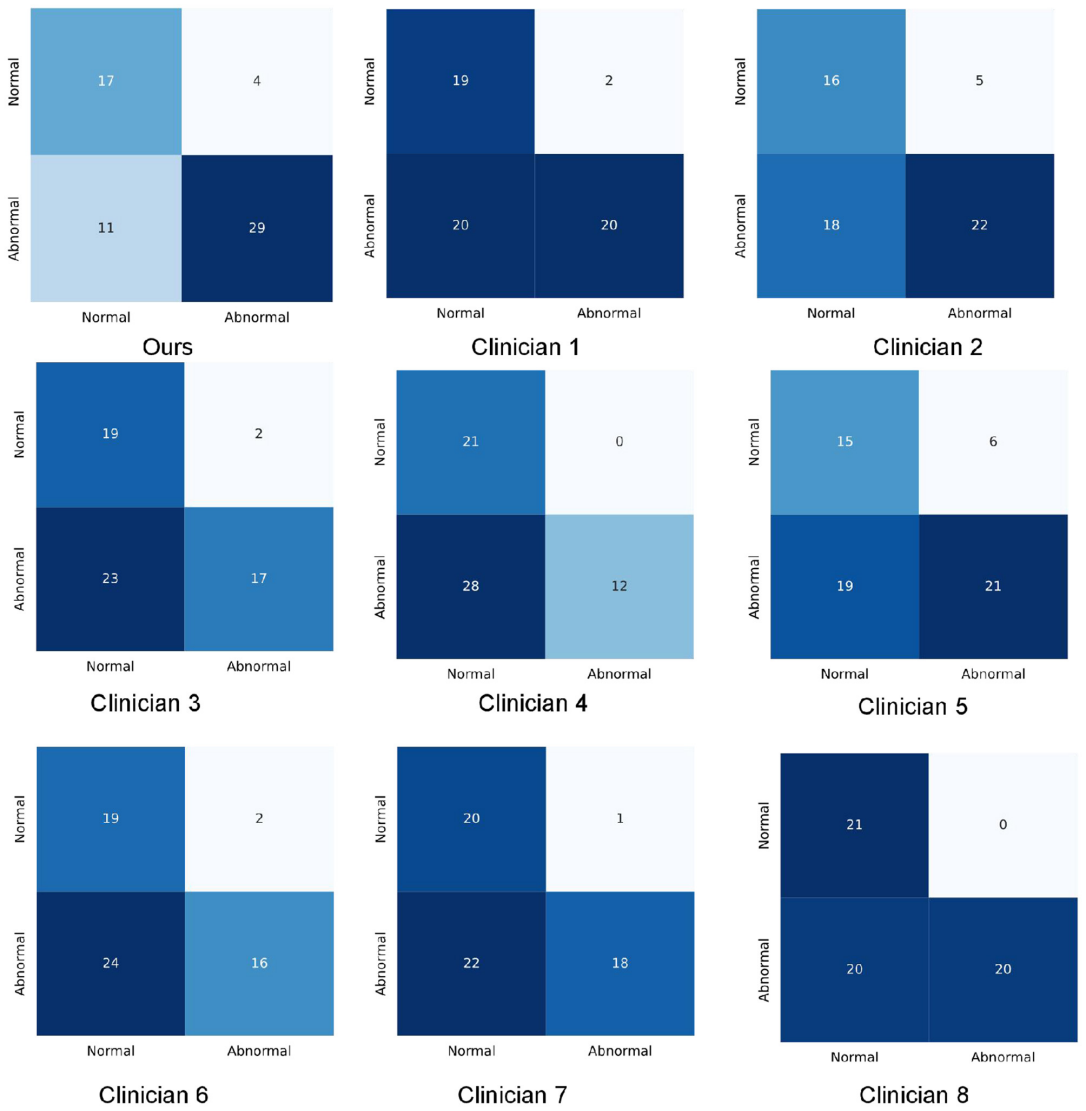
Confusion matrixes of the proposed method and clinicians.

We compared the performance of the deep learning model against neurology specialists on the test dataset. The results demonstrated that the deep learning model outperformed specialists across multiple metrics (Table 2). Specifically, the model achieved an F1 score of 78.94 (95% CI, 67.7–86.4), surpassing the specialists, whose scores ranged from 43.92 to 66.26 (mean: 59.37). Supplementary Figure 4 presents the ROC curves. The model achieved an AUC of 0.842 (95% CI, 0.758–0.921), sensitivity of 71.55% (95% CI, 60.6–85.0%), and accuracy of 74.52% (95% CI, 63.9–83.2%). In contrast, specialist performance yielded a mean AUC of 0.677 (range: 0.630–0.751), mean sensitivity of 45.48%, and mean accuracy of 60.41%. The consistency rate among specialists ranged from 0.61 to 0.87, while their agreement with the model ranged from 0.51 to 0.59. Detailed prediction results are presented in the confusion matrices (Figure 2).

**Table 2:** Comparison results of CTNet and clinicians (95% CI).

**Evaluator**	**Accuracy**	**Sensitivity**	**PPV**	**Specificity**	**F1**	**AUC**
CTNet	**74.52 (63.9–83.2)**	**71.55 (60.6–85.0)**	87.48 (79.5–96.7)	80.29 (63.0–94.2)	**78.94 (67.7–86.4)**	**84.2 (75.8–92.1)**
Clinician 1	64.00 (52.5–73.8)	48.90 (30.6–61.9)	91.87 (80.6–100.0)	90.29 (77.3–100.0)	65.22 (54.2–74.5)	71.10 (60.1–84.0)
Clinician 2	60.85 (47.5–71.8)	56.80 (42.5–71.9)	82.41 (72.7–92.7)	78.00 (58.2–90.5)	66.10 (54.1–79.9)	65.98 (57.0–79.4)
Clinician 3	56.89 (49.5–63.9)	44.30 (23.6–62.5)	89.64 (78.7–100.0)	92.57 (85.7–100.0)	56.26 (42.0–68.5)	66.75 (59.1–75.1)
Clinician 4	52.69 (45.9–61.9)	28.30 (15.0–41.9)	**100.0 (100.0–100.0)**	**100.0 (100.0–100.0)**	43.92 (30.6–59.6)	65.02 (57.5–74.7)
Clinician 5	60.82 (49.5–71.8)	51.75 (38.1–64.4)	78.17 (65.9–90.0)	70.19 (52.4–85.7)	62.56 (47.9–75.0)	62.95 (53.0–74.6)
Clinician 6	58.03 (47.9–68.5)	39.55 (25.0–56.4)	89.00 (75.0–100.0)	89.62 (76.2–100.0)	54.89 (39.6–68.5)	64.59 (56.8–72.1)
Clinician 7	62.75 (52.5–72.1)	43.35 (32.5–60.0)	95.05 (85.9–100.0)	94.67 (85.7–100.0)	59.77 (42.3–71.0)	69.73 (61.8–77.2)
Clinician 8	67.21 (57.7–76.7)	50.90 (42.5–63.9)	**100.0 (100.0–100.0)**	**100.0 (100.0–100.0)**	66.26 (52.5–78.8)	75.12 (67.5–81.0)
Average (clinician)	60.41	45.48	90.77	80.67	59.37	67.66

Bold values indicate the best performance for each metric.

### Comparison with other models

Prior to our study, there was no existing research employing NCCT for HT prediction, rendering a comparative model analysis unfeasible. Earlier independent works by Wozniak et al. (2023) and colleagues, as well as Soundari et al. (2022) and associates, utilized NCCT in the detection of brain tumors and lung cancer respectively. In our study, we have replicated these aforementioned models, employing them for HT prediction using NCCT. The predictive outcomes are presented in Table 3.

**Table 3:** Comparison results of CTNet and other models.

**Model**	**Accuracy**	**Sensitivity**	**Specificity**	**F1**
CTNet	**74.52**	71.55	**80.29**	**78.94**
Marcin Woz nia	64.52	100.00	0.00	39.22
Soundari D V	64.52	100.00	0.00	39.22

Bold values indicate the best performance for each metric.

## Discussion

This study demonstrates the feasibility of using deep learning to predict HT after ischemic stroke, identifying potential risks even under conditions of complex and limited information. This tool aims to assist clinicians in individualized diagnosis, risk stratification, and the management of high-risk patients. Our model outperformed physicians in accuracy when utilizing only image information, presenting a novel approach for clinical HT prediction.

Unlike studies utilizing perfusion MRI (Yu et al., 2018), our approach employs NCCT, which is more accessible, faster, and widely applicable. While Dharmasaroja and Dharmasaroja (2012) achieved an AUC of 0.787 using machine learning on clinical data, a gap remains compared to CTNet's performance. Furthermore, while previous imaging studies focused on local features (Strbian et al., 2012; Whiteley et al., 2012; Bang et al., 2011; Kidwell et al., 2008), our deep learning approach analyzes complete CT images, allowing the network to learn a broader range of radiological features and their complex interconnections. Due to the unavailability of comprehensive clinical data, we were unable to assess clinical scale scores for our dataset. However, a multicenter trial of 3,035 AIS patients evaluated various scales for symptomatic HT, reporting AUCs ranging from 0.68 (IST-3) to 0.56 (SPAN-100) (Whiteley et al., 2014). In comparison, CTNet achieved an AUC of 0.842 (95% CI: 0.758–0.921), demonstrating superior predictive capability.

**Figure 3:**
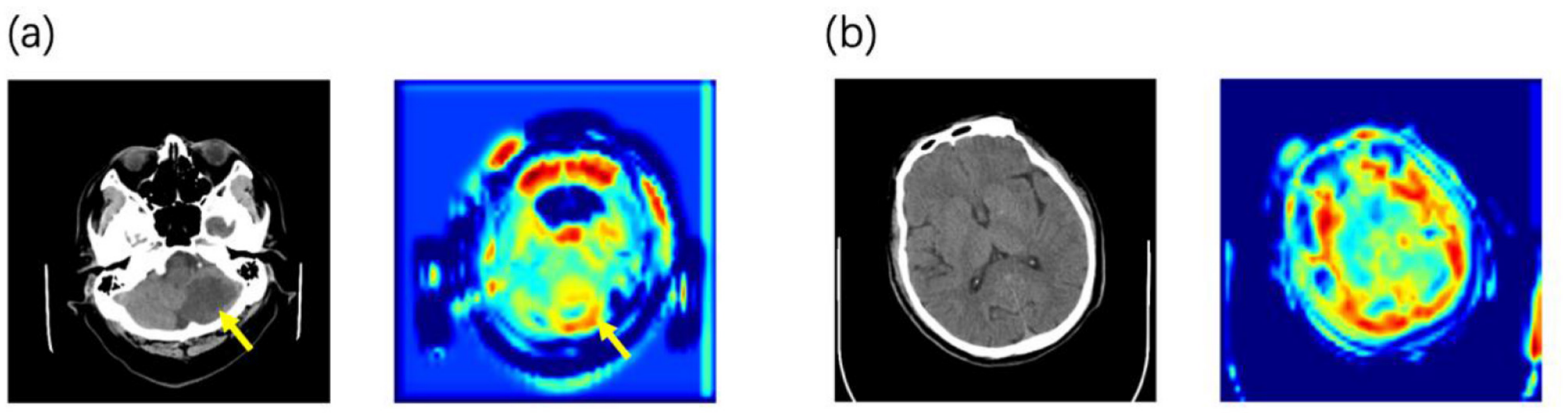
Examples of the predicted NCCT scan images alongside its corresponding attention heatmaps **(a)** When lesions are detectable on CT, the model focuses its attention on the lesion area. **(b)** In the early stage before lesions are visible on CT, the model's attention is distributed across the entire brain parenchyma.

Using attention heatmaps, we visualized the anatomical regions utilized by the model. In cases with visible lesions (Figure 3a), the algorithm's focus correlated with the infarct area, confirming that the model effectively prioritized relevant pathological features. Notably, the model outperformed specialists in cases with low lesion visibility (Figure 3b), where it attended to specific brain regions that doctors might overlook. This suggests that deep learning attention maps could uncover subtle early indicators of HT.

Deep neural networks (DNNs) have demonstrated significant potential in predicting hemorrhagic transformation (HT) following ischemic stroke, owing to their capacity to extract intricate features from extensive medical imaging datasets. Nevertheless, the current model requires further training and optimization to meet the rigorous demands of clinical application. Specifically, future research should prioritize enhancing the model's accuracy and generalizability by integrating heterogeneous data sources and refining the network architecture. Furthermore, validating the model within real-world clinical workflows is imperative to ascertain its feasibility and effectiveness in supporting decision-making. Ultimately, the development of robust and precise deep learning frameworks for HT prediction holds the promise of improving stroke management through earlier detection and personalized treatment strategies, thereby optimizing both short- and long-term patient outcomes.

## Limitations of the study

Despite exhibiting promising performance, our current work still has some limitations. Firstly, due to the limited sample size, we were unable to stratify patients according to their different treatment plans, which can have a significant impact on the occurrence of HT. Secondly, we did not perform segmentation of the lesion area, as we considered that unknown features outside the lesion area may also contribute to the prediction accuracy. In future studies, it may be worth investigating the use of lesion segmentation to further enhance the model's predictive performance. Thirdly, we have yet to establish a model built on a multi-center database, which may lead to limitations in the generalization ability of our model. Our future objective is to develop a model that can incorporate multi-modal data. This will require integrating a comprehensive and easily obtainable set of clinical data with imaging data to improve the accuracy and robustness of our model. Moreover, in analyzing the heatmap of attention, we observed that the model displayed attention toward areas corresponding to the skull, potentially affecting its performance. This indicates a need to remove the skull during image preprocessing. Ultimately, we aim to apply this model in a clinical setting to provide individualized treatment plan support and assist in decision-making for each patient.

## Data Availability

The raw data supporting the conclusions of this article will be made available by the authors, without undue reservation.
