# Supplementary material for: Neurologists-level interpretable CT-based deep neural network for prediction of hemorrhagic transformation after ischemic stroke
Source: Front Neurosci. 2026 Jan 14;19:1753071. doi: 10.3389/fnins.2025.1753071 (PMC12847036; doi:10.3389/fnins.2025.1753071)
Supplement: Supplementary file 1 [file Data_Sheet_1.docx]

**Supplementary Information**

**Supplementary Figure 1:** Data pre-processing process

**Supplementary Figure 2:** CNN and Residual blocks.

**Supplementary Figure 3:** The architecture of CTNet

**Supplementary Figure 4:** ROC curve of CTNet and AUC values of CTNet and doctors.
